# Long-term effects of low-fat diets either low or high in protein on cardiovascular and metabolic risk factors: a systematic review and meta-analysis

**DOI:** 10.1186/1475-2891-12-48

**Published:** 2013-04-15

**Authors:** Lukas Schwingshackl, Georg Hoffmann

**Affiliations:** 1 Department of Nutritional Sciences, Faculty of Life Sciences, University of Vienna, Althanstraße 14 (UZII), Vienna, A-1090, Austria

**Keywords:** High-protein, Cardiovascular risk factors, Low-fat, Glycemic control

## Abstract

**Background:**

Meta-analyses of short-term studies indicate favorable effects of higher protein vs. lower protein diets on health outcomes like adiposity or cardiovascular risk factors, but their long-term effects are unknown.

**Methods:**

Electronic databases (MEDLINE, EMBASE, Cochrane Trial Register) were searched up to August 2012 with no restriction to language or calendar date. A random effect meta-analysis was performed using the Software package by the Cochrane Collaboration Review Manager 5.1. Sensitivity analysis was performed for RCTs with a Jadad Score ≥3, and excluding type 2 diabetic subjects (T2D).

**Results:**

15 RCTs met all objectives and were included in the present meta-analysis. No significant differences were observed for weight, waist circumference, fat mass, blood lipids (i.e. total cholesterol, LDL-cholesterol, HDL-cholesterol, triacylglycerols), C-reactive protein, diastolic and systolic blood pressure, fasting glucose and glycosylated hemoglobin. In contrast, improvements of fasting insulin was significantly more pronounced following high protein diets as compared to the low protein counterparts (weighted mean difference: -0.71 μIU/ml, 95% CI -1.36 to -0.05, p = 0.03). Sensitivity analysis of high quality RCTs confirmed the data of the primary analyses, while exclusion of studies with diabetic subjects resulted in an additional benefit of high-protein diets with respect to a more marked increase in HDL-cholesterol.

**Conclusion:**

According to the present meta-analysis of long-term RCTs, high-protein diets exerted neither specific beneficial nor detrimental effects on outcome markers of obesity, cardiovascular disease or glycemic control. Thus, it seems premature to recommend high-protein diets in the management of overweight and obesity.

## Background

With respect to the optimal macronutrient composition in the daily diet, most international authorities recommend to increase intakes of carbohydrates at the expense of fat and protein [1,2]. However, in face of the worldwide increase in prevalence of both overweight and obesity, there is a plethora of recommendations for diets aiming at weight loss and weight management. Among them, a high-protein (HP) regimen has gained increasing interest in recent years [3]. For the general population, recommended dietary reference intakes (DRIs) for protein are 0.66 g * kg body weight^-1^ * d^-1^[4]. Actual consumption data for the US American population average 1.3 g * kg body weight^-1^ * d^-1^ in the 19–30 age group indicating a protein intake in excess of their needs [5]. The Acceptable Macronutrient Distribution Range (AMDR) for protein is given as 5-35% of daily calories depending on age [6]. A recent meta-analysis comparing HP vs. low-protein (LP) diets with a duration between 28 days and 12 months observed favorable effects of HP diets on biomarkers of obesity as well as cardiovascular risk factors such as HDL-cholesterol (HDL-C), triacylglycerols (TG), and blood pressure [7]. Several randomized controlled trials (RCTs) investigated the short-term effects of HP vs. LP diets, reporting advantages of HP protocols including a reduction in TG concentration [8-10]. A meta-regression of 87 studies concluded that low-carbohydrate, HP diets favorably affected body mass and composition independent of energy intake [11]. The benefits of HP diets might be explained by increased thermogenesis and satiety [12,13]. Recent data from the 26-year follow up of the Nurses’ Health Study (NHS) revealed that protein sources such as red meat and high-fat dairy products were significantly associated with an elevated risk of coronary heart disease, while higher intakes of poultry, fish, and nuts correlated with a lower risk of coronary heart disease (CHD) [14]. Since there is a lack of information concerning studies with different protein contents covering a longer dietary intervention period, the aim of this meta-analysis was to compare the long-term effects of HP vs. LP regimens on biomarkers of obesity, cardiovascular complications as well as adverse effects of HP.

## Methods

The review protocol has been registered in PROSPERO International Prospective Register of Systematic Reviews (crd.york.ac.uk/prospero/index.asp Identifier: CRD42012002791).

### Literature search

Literature search was performed using the electronic databases MEDLINE (between 1966 and August 2012), EMBASE (between 1980 and August 2012), and the Cochrane Trial Register (until August 2012) with restrictions to randomized controlled trials, but no restrictions to language and calender date using the following search term: *(high protein diet).* Moreover, the reference lists from retrieved articles were checked to search for further relevant studies. This systematic review was planned, conducted, and reported adhearing to standards of quality for reporting meta-analyses [15]. Literature search was conducted independently by both authors, with disagreements resolved by consensus.

### Eligibility criteria

Studies were included in the meta-analysis if they met all of the following criteria: (1) randomized controlled design; (2) minimum intervention period with a follow-up of 12 months; (3) comparing a HP (≥ 25% of total energy content, TEC) with a LP dietary intervention (≤ 20% of TEC), with both protocols adopting a low fat diet (≤ 30% of TEC) [16]; (4) assessment of the outcome markers: weight, waist circumference (WC), fat mass (FM), total cholesterol (TC), low-density lipoprotein cholesterol (LDL-C), HDL-C, TG, diastolic and systolic blood pressure (DBP, SBP), C-reactive protein (CRP), fasting glucose (FG), fasting insulin (FI) and glycosylated hemoglobin (HbA1c); (5) report of post-intervention mean values (if not available mean of two time points were used) with standard deviation (or basic data to calculate these parameters). If data of ongoing studies were published as updates, results of only the longest duration periods were included.

### Quality assessment of studies

**Figure 1:**
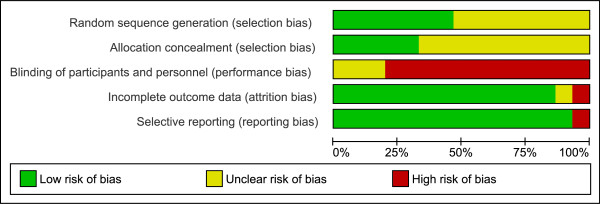
**Risk of bias assessment tool.** Across trials, information is either from trials at a low risk of bias (green), or from trials at unclear risk of bias (yellow), or from trials at high risk of bias (red).

Full copies of studies were independently assessed for methodological quality by both authors using the Jadad score [17]. This 5-point quality scale includes points for randomization (randomized = 1 point; table of random numbers or computer generated randomization = an additional 1 point), double-blinding (double-blind = 1 point; use of a placebo = additional 1 point), and follow-up (numbers and reasons for withdrawal in each group are stated = 1 point) within the report of an RCT. An additional point was accepted if the analysis was by intention-to-treat to compensate for the fact that double-blinded study protocols are elusive in dietary intervention studies. Final scores of 0–2 were considered as low quality, while final scores of ≥ 3 were regarded as representing studies of high quality. Furthermore, the trials were assessed for methodological quality using the risk of bias assessment tool by the Cochrane Collaboration [18] (Figure 1).

### Data extraction and statistical analysis

The following data were extracted from each study: the first author’s last name, publication year, study duration, participant´s sex and age, BMI, % diabetics, sample size, outcomes, drop outs and post mean values or differences in mean of two time point values with corresponding standard deviation. Subsequently, a standardized data extraction form for this systematic review was created according to Avenell et al. [19]. For each outcome measure of interest, a meta-analysis was performed in order to determine the pooled effect of the intervention in terms of weighted mean differences (WMDs) between the post-intervention (or differences in means) values of the HP and LP groups. Combining both the post-intervention values and difference in means in one meta-analysis is a legitimate method described by the Cochrane Collaboration [20]. All data were analyzed using the REVIEW MANAGER 5.1 software, provided by the Cochrane Collaboration (http://ims.cochrane.org/revman). Heterogeneity between trial results was tested with a standard χ^2^ test. The I^2^ parameter was used to quantify any inconsistency: I^2^ = [(Q – d.f.)]/Q × 100%, where Q is the χ^2^ statistic and d.f. is its degrees of freedom. A value for I^2^ > 50% was considered to represent substantial heterogeneity [21]. To consider heterogeneity, the random-effects model was used to estimate WMDs with 95% confidence intervals (CIs). Forest plots were generated to illustrate the study-specific effect sizes along with a 95% CI. Funnel plots were used to assess potential publication bias (e.g. the tendency for studies that yield statistically significant results to be more likely to be submitted and accepted for publication). To determine the presence of publication bias, the symmetry of the funnel plots in which mean differences were plotted against their corresponding standard errors was assessed. One study [22] imputed two types of LP diets, and these diets were combined to one group as described in the Cochrane Handbook [20]. Data extraction was conducted independently by both authors, with disagreements resolved by consensus.

## Results

### Literature search and characteristic of studies

**Figure 2:**
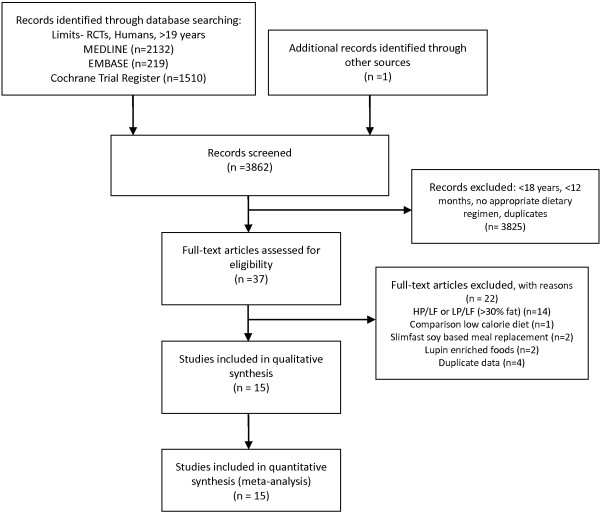
Flow diagram.

A total of 15 studies extracted from 3862 articles met the inclusion criteria and were analyzed in the systematic review [22-36]. The detailed steps of the meta-analysis article selection process are given as a flow chart in Figure 2. General study characteristics are given in Table 1. In case of more than one LP/LF group within a single study design, all LP interventions were combined as recommended by the Cochrane Collaboration [20]. Type 2 diabetes mellitus (T2D) was not defined as an exclusion criteria, and a total of three studies enrolling subjects with T2D were included in the present meta-analysis [24,32,33]. 13/15 studies reported the distribution of gender (1200 women vs. 690 men).

**Table 1:** General characteristics of randomized controlled intervention trials included in the meta-analysis

**Reference**	**Sample size,**	**Age,**	**Duration, months**	**Dietary intervention**	**Dietary protocol**	**Energy restricted (kcal)**	**Drop Out**	**Study quality**
	**BMI (kg/m**^**2**^**),**	**Female (%)**			**Protein(%), Carbohydrates(%), Fat(%)**			
	**% diabetics**	**Male (%)**						
Brinkworth et al. 2004 I [23]	58	50.2	16	HP/LF vs.	30%, 40%, 30%	1555 (12 weeks), energy balance (4 weeks), no restriction (follow up)	27%	2
	34	77.5%		LP/LF	15%, 55%, 30%	1555 (12 weeks), energy balance (4 weeks), no restriction (follow up)	23%	
	0%	22.5%						
Brinkworth et al. 2004 II [24]	66	>60	15	HP/LF vs.	30%, 40%, 30%	1600 (8 weeks) energy balance (4 weeks), no restriction (follow up)	39%	3
	27-40	n.d		LP/LF	15%, 55%, 30%	1600 (8 weeks) energy balance (4 weeks), no restriction (follow up)	42%	
	100%	n.d						
Clifton et al. 2007 [25]	79	49	15	HP/LF vs.	34%, 46%, 20%	1340 (12 weeks), energy balance (follow up, 52 weeks)	29%	2
	32.8	100%		LP/LF	17%, 64%, 20%	1340 (12 weeks), energy balance (follow up, 52 weeks)	38%	
	0%	0%						
Dansinger et al. 2005 [26]	80	49	12	HP/LF vs.	30%, 40%, 30%	no	35%	4
	35	48%		LP/LF	10-15%, >65%, 10%	no	50%	
	n.d	52%						
Das et al. 2007 [27]	34	35	12	HP/LF vs.	30%, 40%, 30%	1900	18%	2
	27.6	n.d		LP/LF	20%, 60%, 20%	1960	12%	
	0%	n.d						
Delbridge et al. 2009 [28]	141	44	12	HP/LF vs.	30%, 40%, 30%	no	37%	3
	39	50%		LP/LF	15%, 55%, 30%	no	41%	
	n.d	50%						
Due et al. 2004[29]	50	39.6	12	HP/LF vs.	30%, 40%, 30%	no	8%	1
	30.4	76%		LP/LF	15%, 55%, 30%	no	28%	
	0%	24%						
Gardner et al. 2007 [22]	232	40.6	12	HP/LF vs.	30%, 40%, 30%	yes	23%	4
	31.33	100%		LP/LF^*^	10-15%, 55-70%, 10/30%	no/yes	23%	
	0%	0%						
Keogh et al. 2007 [31]	25	48.7	12	HP/LF vs.	40%, 33%, 27%	1435	n.d	1
	32.9	68%		LP/LF	20%, 60%, 20%	1435	n.d	
	0%	32%						
Krebs et al. 2012 [32]	419	57.9	24	HP/LF vs.	30%, 40%, 30%	-500	30%	4
	36.6	60%		LP/LF	15%, 55%, 30%	-500	24%	
	100%	40%						
Larsen et al. 2011 [33]	99	59.2	12	HP/LF vs.	30%, 40%, 30%	1530 (3 months), energy balance (follow up)	19%	4
	27-40	52%		LP/LF	15%, 55%, 30%	1530 (3 months), energy balance (follow up)	20%	
	100%	48%						
Layman et al. 2008 [30]	130	45.4	12	HP/LF vs.	30%, 40%, 30%	1700 women, 1900 men	36%	2
	32.6	55%		LP/LF	15%, 55%, 30%	1700 women, 1900 men	55%	
	n.d	45%						
McAuley et al. 2006 [34]	48	n.d	12	HP/LF vs.	30%, 40%, 30%	no	7%	2
	n.d	100%		LP/LF	15%, 55%, 30%	no	25%	
	Insulin resistant	0%						
Sacks et al. 2009 [35]	406	50.5	24	HP/LF vs.	25%, 55%, 20%	-750	22%	4
	33	64%		LP/LF	15%, 65%, 20%	-750	16%	
	0%	36%						
Wycherley et al. 2012 [36]	123	20-65	12	HP/LF vs.	35%, 40%, 25%	1700	43%	4
	27-40	0%		LP/LF	17%, 58%, 25%	1700	44%	
	0%	100%						

^*^two kind of LP/LF diets (very LF: 10% and LF: 30% of total energy content).

HP, high-protein; LF, low fat; LP, low-protein; n.d, no data.

The pooled estimate of effect size for the effects of HP as compared to LP on primary and secondary outcomes are summarized in Table 2.

**Table 2:** **Pooled estimates of effect size (95**% **confidence intervals) expressed as weighted mean difference for the effects of HP vs. LP diets on cardiovascular and metabolic risk factors**

**Outcomes**	**No. of studies**	**Participants**	**WMD**	**95% CI**	**p-values**	**Inconsistency I**^**2**^
Weight (kg)	13	971	-0.39	[-1.43, 0.65]	0.46	0%
WC (cm)	8	727	-0.98	[-3.32, 1.37]	0.41	72%
FM (kg)	10	913	-0.59	[-1.32, 0.13]	0.11	0%
TC (mg/dl)	12	1251	-2.51	[-7.74, 2.71]	0.35	32%
LDL-C (mg/dl)	13	1522	1.58	[-5.36, 8.53]	0.66	79%
HDL-C (mg/dl)	14	1563	0.90	[-0.09, 1.89]	0.08	0%
TG (mg/dl)	14	1563	-2.87	[-11.13, 5.38]	0.49	21%
DBP (mmHg)	11	1402	-0.42	[-1.37, 0.54]	0.39	0%
SBP (mmHg)	11	1414	-1.61	[-3.45, 0.23]	0.09	41%
CRP (mg/dl)	4	222	0.22	[-0.36, 0.79]	0.46	0%
FG (mg/dl)	11	1357	-0.63	[-1.93, 0.67]	0.34	0%
FI (μIU/ml)	11	1086	-0.71	[-1.36, -0.05]	**0.03**	0%
HbA1c (%)	3	431	0.07	[-0.17, 0.31]	0.55	0%

CI, confidence intervalls, CRP, high-sensitive-C reactive protein; DBP, diastolic blood pressure; FG, fasting glucose; FI, fasting insulin; FM, fat mass; HbA1c, glycosylated hemoglobin; HDL-C, high-density lipoprotein cholesterol; LDL-C, low-density lipoprotein cholesterol; SBP, systolic blood pressure; TC, total cholesterol, TG, triacyglycerols; WC, waist circumference; WMD, weighted mean difference.

### Data analysis

#### Weight/ Waist circumference/ Fat Mass

Weighted mean differences (WMD) in change of weight [-0.39 kg (95% CI -1.43 to 0.65), p = 0.46], WC [-0.98 cm (95% CI -3.32 to 1.37), p = 0.41] and FM [-0.59 kg (95% CI - -1.32 to 0.13), p = 0.11] were not statistically significant when comparing HP vs. LP dietary protocols.

#### Serum lipids

No significant changes were observed for TC [WMD: - 2.51 mg/dl (95% CI -7.74 to 2.71), p = 0.35], LDL cholesterol [WMD: 1.58 mg/dl (95% CI -5.36 to 8.53), p = 0.66], HDL cholesterol [WMD: 0.90 mg/dl (95% CI -0.09 to 1.89), p = 0.08], and TG [WMD: -2.87 mg/dl (95% CI -11.13 to 5.38), p = 0.49] between HP and LP diets.

#### Blood Pressure/CRP

When comparing HP and LP regimen, no significant differences could be found with respect to WMDs in change of CRP [0.22 mg/dl (95% CI -0.36 to 0.79), p = 0.46] and blood pressure values [DBP: -0.42 mmHg (95% CI -1.37 to 0.54), p = 0.39; and SBP: -1.61 mmHg (95% CI -3.45 to 0.23), p = 0.09].

#### Glycemic control

**Figure 3:**
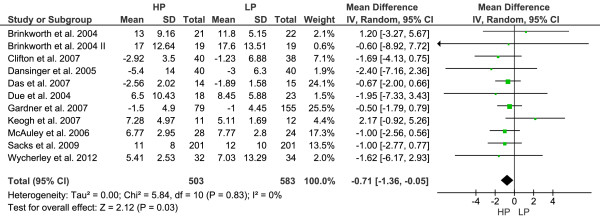
**Forest plot showing pooled WMD with 95% CI for fasting insulin (μIU/ml) for 10 randomized controlled high-protein diet studies.** For each high-protein study, the shaded square represents the point estimate of the intervention effect. The horizontal line joins the lower and upper limits of the 95% CI of these effects. The area of the shaded square reflects the relative weight of the study in the respective meta-analysis. The diamond at the bottom of the graph represents the pooled WMD with the 95% CI for the 10 study groups. Abbreviations: HP = high-protein; LP = low-protein; I^2^ = Inconsistency.

Decreases in FI were significantly more explicit in subjects adhering to an HP diet as compared to those following an LP regimen [WMD: -0.71 μIU/ml (95% CI -1.36 to -0.05), p = 0.03] (Figure 3). None-significant changes were observed for FG [WMD: -0.63 mg/dl (95% CI -1.93 to 0.67), p = 0.34], and HbA1c [WMD: 0.07% (95% CI -0.17 to 0.31), p = 0.55].

### Sensitivity analysis

**Figure 4:**
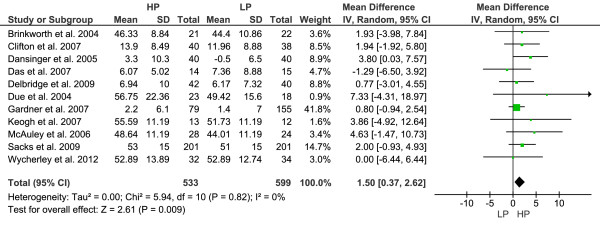
**Forest plot showing pooled WMD with 95% CI for HDL-cholesterol (mg/dl) for 10 randomized controlled high-protein diet studies (excluding T2D subjects).** For each high-protein study, the shaded square represents the point estimate of the intervention effect. The horizontal line joins the lower and upper limits of the 95% CI of these effects. The area of the shaded square reflects the relative weight of the study in the respective meta-analysis. The diamond at the bottom of the graph represents the pooled WMD with the 95% CI for the 10 study groups. Abbreviations: HP = high-protein; LP = low-protein; I^2^ = Inconsistency; T2D = type 2 diabetes.

Articles with a Jadad quality score ≥ 3 only were included in the sensitivity analyses. A total of 8/15 studies remained for sensitivity analyses [22,24,26,28,32,33,35,36]. The results of the primary analyses could be confirmed for all the parameters that were not significantly altered in different ways in the HP and LP groups. Furthermore, changes in FI turned out to be of similar dimension as well when studies with poor Jadad scores were excluded. In an additional sensitivity analysis, studies enrolling patients with T2D [33,34,36] were discarded to account for a potential “reproducibility effect” on the pooled WMD when comparing the results of the present systematic review with a meta-analysis by Santesso et al. [7], where T2D represented an exclusion criterium. Results were not significantly different as compared to the comprehensive meta-analyses except for one parameter, i.e. the beneficial increase in HDL-C was more pronounced in the HP as compared to the LP diet [WMD: 1.50 mg/dl (95% CI 0.37 to 2.62), p = 0.009] (Figure 4).

### Publication bias

The funnel plots (with respect to effect size changes for Weight, WC, FM, TC, LDL-C, HDL-C, TG, CRP, DBP, SBP, FG, FI and HbA1c in response to HP diets) indicates little to moderate asymmetry, suggesting that publication bias cannot be completely excluded as a factor of influence on the present meta-analysis. It remains possible that small studies yielding inconclusive data have not been published.

### Heterogeneity

Considerable heterogeneity was found with respect to WC (I^2^ = 72%) and LDL-C (I^2^ = 79%) in the primary analysis. Moderate heterogeneity was observed for TC (I^2^ = 32%) and SBP (I^2^ = 41%), the other outcomes showed low heterogeneity (I^2^ = 0-21%).

## Discussion

In this systematic review, HP dietary protocols were compared with LP regimens with respect to their effects on biomarkers of obesity and obesity-associated disorders such as diabetes or cardiovascular disease. Analyses were restricted on HP as well as LP diets providing ≤ 30% of TEC in the form of fat to prevent potential bias due to variations in total fat intake. Main findings suggest no advantages or disadvantages of a higher dietary protein content. None of the dietary protocols turned out to be superior to its counterpart with regard to the biomarkers under investigation. Following primary analysis, decreases in fasting insulin were significantly more pronounced in HP diets. However, this was no longer valid after inclusion of high quality trials only in the secondary analysis. The raise in HDL-C turned out to be more pronounced in the HP group compared to the LP group following sensitivity analysis excluding studies that enrolled patients with T2D. In a previous study, HP diets exerted a 12%-increase in HDL-C under closely supervised dietary control [37]. Two meta-analyses provide evidence that higher fat intake was associated with higher levels of HDL-C when compared to low-fat diets [38,39]. With respect to the studies included in the present systematic review, the trials by Gardner et al. [22], Dansinger et al. [26], and McAuley et al. [34] reported higher intakes of total fat at the end of their 12 months protocols (dietary records) in the HP groups as compared to the respective LP counterparts. Omitting these trials to the sensitivity analysis, changes in HDL-C turned out to be similar in both HP and LP regimen (data not shown), suggesting that HDL-C response was due to dietary fat content rather than to protein consumption.

Taken together, these results are in discrepancy with a recent meta-analysis by Santesso and co-workers [7] who reported weight loss, WC, HDL-C, TG, SBP, DBP and FI to be significantly more improved following short-and long-term HP diets as compared to LP protocols. The different findings might at least in part be explained by the fact that only long-term studies with a duration ≥ 12 months were included in the present meta-analysis. In addition, both post-intervention values as well as changes in mean differences were used as suggested by the Cochrane Collaboration [20] to avoid a standardized mean differences method, whereas Santesso et al. [7] separated between primary (change from baseline values) and secondary (final values) analyses. These results indicate that HP diets do not exert favorable effects on anthropometric measures like body weight, fat mass and waist circumference. However, in a meta-regression by Krieger et al. [11] high-protein intake turned out to be a significant predictor of fat free mass retention, thereby compensating a potential side-effect of long-term energy restriction.

Dietary protein content of the high-protein diets included in this meta-analysis varied between 30-40% of TEC, which is within the age-dependent AMDR of 5-35% for all but one RCT [31]. Via analysis of the National Health and Nutrition Examinations Survey conducted between 2003 and 2004, Fulgoni [5] concluded that the actual intake of protein in US-American adults of 1.3 g * kg body weight^-1^ * d^-1^ exceeds the DRI values of 0.66 g * kg body weight^-1^ * d^-1^. He suggested that recommendations could be adapted to 25-30% of TEC, assuming benefits of higher protein intake e.g. on regulation of body weight. Regarding biomarkers such as weight, waist circumference or fat mass, the present meta-analysis does not support this concept.

Three RCTs included in this meta-analysis investigated the effects of HP regimens on biomarkers of kidney function in patients with T2D. In all trials, HP diets did not affect renal functions assessed via measurement of serum creatinine and microalbuminurea [32,33,36]. Likewise, a 2-year RCT by Friedman et al. [40] reported no harmful effects of a high-protein/low carbohydrate diet on glomerular filtration rate, albuminuria, or fluid and electrolyte balance. With respect to prospective cohort studies, a systematic review by Mente et al. [41] indicated no significant correlations between animal protein sources, e.g. eggs, milk or meat on coronary heart disease (CHD), whereas vegetable protein sources like nuts were associated with a decreased risk. Findings from Greece, Sweden and the US noted an increased all-cause mortality following a HP/low carbohydrate diet based on animal sources in both women and men whereas a vegetable-based low-carbohydrate diet was associated with lower all-cause and cardiovascular disease mortality rates [42-44].

This systematic review did not consider unpublished data, and with respect to the moderate asymmetry of the Funnel plots, it cannot be excluded that publication bias such as lack of published studies with inconclusive results may have at least a moderate impact on the effect size estimates. An important limitation of dietary intervention trials is the heterogeneity of various aspects and characteristics of the study protocols. The literature chosen for the present meta-analysis varies regarding type(s) of diets used, definitions of HP and LP diets, study population (i.e. BMI, type 2 diabetics, abnormal glucose metabolism), intervention time, nutritional assessment as well as long-term follow-ups (between 1 and 2 yrs.). In addition, some studies were performed on hypocaloric terms, while others provided an isocaloric diet.

Not all of the studies gave details on the quality of their respective setup (e.g. method of randomization, follow-up protocol with reasons for withdrawal) yielding Jadad scores < 3. However, following sensitivity analyses including high quality studies only (Jadad score ≥ 3), pooled estimates of effect size were similar to those obtained with the complete set of studies. Some comparisons within the present meta-analyses were done using both post-intervention values and changes in mean difference, which is considered to be a legitimate procedure as described by the Cochrane Collaboration [20], and should not be regarded as a limitation.

In summary, the present meta-analysis investigated the long-term effects of HP vs. LP both low in fat on biomarkers predicting the outcome of obesity, cardiovascular disease and glycemic control. Since biomarkers under investigation were not affected by changes in dietary protein content, unanimous recommendation of a high-protein dietary approach is not supported by evidence. With respect to the potential risk of high-protein contents, further studies are required before dietary recommendations can be changed towards a higher percentage of daily protein consumption.

## Competing interests

Both Author declare that they have no competing interest.

## Authors’ contributions

LS and GH conducted the data analysis, interpretation of results, manuscript drafting, and finalizing manuscript. All authors read and approved the final manuscript.
